# Abstracting It All: The Soviet Institute of Scientific Information (VINITI) and the Promise of Centralisation, 1952–1977

**DOI:** 10.1007/s11024-024-09545-z

**Published:** 2024-09-26

**Authors:** Björn Hammarfelt, Johanna Dahlin

**Affiliations:** 1 Swedish School of Library and Information Science, University of Borås, Borås, Sweden; 2 Department of Culture and Society, Linköping University, Norrköping, Sweden

**Keywords:** Academic publishing, Scholarly communication, Soviet, Scientific information

## Abstract

In the aftermath of the Second World War, effective handling of scientific information was identified as crucial for advancement and international competitiveness. Here, we study how the Soviet Union, through the founding of *The All-Union Institute for Scientific and Technical Information* (VINITI), developed its own grandiose system which served researchers and engineers throughout the USSR. By studying its inception, the way it was structured, and how it relates to similar grand visions of how to organise knowledge, we provide rare insights into a partly alternative history of how scientific information was organised in the latter half of the 20^th^ century. Based on available sources in English and Russian, we consider the ideas behind this grand initiative for acquiring international literature, as well as how it was received and presented to a foreign audience. In this effort, we put particular emphasis on the first 25 years of VINITI (1952–1977) while at the same time focusing on central ideas in its organisation such as “enrichment”, “abstracting” and “pre-printing”. A key principle emerging from our analysis is how the notion of concentration becomes a fundamental principle for its operations. Overall, the activities of VINITI can today appear as both old-fashioned, bordering on the utopian, and as visionary and modern in its abandonment of journals and traditional forms of peer review.

## Introduction

On the 19^th^ of July 1952, the All-Union Institute for Scientific and Technical (Information Vsesoyuznyi Institut Nauchnoi Tekhnicheskoi Informatsii in Russian), initially named the Institute for Scientific Information, was launched by the USSR Academy of Sciences. Known under the acronym VINITI (which we will use henceforth), the institute was founded on the initiative of the chemist, and then president of the Soviet Academy of Sciences, Alexander Nesmeyanov (1899–1980). Already from the start, VINITI was a large initiative and in time it came to live up to the epithet colossal: in the 1980s, it is estimated that it employed over 26,000 people, of which 2,300 were science writers and 2,000 translators. It handled 38,000 journals with 1,2 million articles yearly (Semeria 1987 cited in Schneider 1994: 168). In his autobiography, Nesmeyanov described his vision at the time: “I envisioned the Institute for Scientific Information as a colossal enrichment plant (concentrator), which would sift through all scientific periodicals of the world” and on this basis produce abstract journals (Nesmeyanov 1999: 54).

The global ambitions of the institute resonated beyond the borders of the Soviet Union, and the news of a large, centralised information centre was greeted with both curiosity and concern by American scientists and policymakers. In an editorial to *Biological Abstracts* 1957 it was claimed that Sputnik might have been more spectacular yet it was the establishment of VINITI that would have the greater impact on science both in Russia and the world (Chernyi 2005: 34). Similarly, *The Washington Post* gave in 1960 an admiring description of what was considered to be “the world largest scientific library.” (Tell 1990: 34). VINITI represented an ambition to gather all technical and scientific information in one place. For librarians and information specialists working in Western countries in which scientific and technological information was dispersed among many different actors, the notion of an all-encompassing institute appeared both tempting and threatening. Indeed, in detailing the history of the American Documentation Institute, Farkas-Conn (1990: 164) claims that the organisation did not “receive foundation support until the late 1950s, when Sputnik, the first space satellite launched by the Soviet Union, sparked life into national science and scientific communication”. Similarly, Trembach (2016: 12) suggests that one of the chief concerns of information specialist was the vast amount of material being printed, with the suspicion being that the centralised solution of the Soviet Union might be better equipped to handle the rapid increase in information.

So, from its inception, VINITI generated curiosity among foreign onlookers. For example, it clearly inspired Eugene Garfield who simply named his company, Institute of Scientific Information (ISI), after its Soviet counterpart with the goal of creating a service that would do the same service, but with less resources (Trembach 2016: 91, Aronova 2021: 139). By outlining the early history of VINITI from its inception in 1952, we show how the development of a Soviet institute for scientific information could be placed in a longer tradition of utopian thinking on the organisation of scientific knowledge. More specifically, we consider the main ideas behind its construction and organisation: what were the visions behind its foundation and the services it provided, and how may we understand these ideas in relation to the broader international context of utopian thinking in relation to scientific information? When answering these questions, we will focus on three key ideas behind the organisation of VINITI. The first section focuses on the creation of the centre in 1952 and the central idea of “concentration”, the second part analyses the role of “abstracting” as a key feature in the operations of VINITI, while the third section covers the practice of “pre-printing” or what was then called “deposition of scientific works” as an innovative feature of the Soviet solution to the problem of scientific information.

Our approach is inspired by Aronova’s use of “Russia as method”, as an anchoring point from which to approach issues of global significance. The phrase is adapted from the concept of “Asia as method” and is an attempt to use a region strategically as a methodological opportunity, to de-universalise histories. Aronova suggests using “Russia as an anchoring point that reveals the circulation, appropriation and modification of knowledge, practices, and philosophies associated with scientific history” (Aronova 2021: 7). It is a geographic lens from which to consider issues of scientific information that were preoccupying scholars in many countries, and ideas that were circulating widely globally. We have chosen VINITI as a case from which to consider the Soviet solution to the information problem and to illuminate certain ideas with a global reach from a certain vantage point. By de-universalising ideas, we can point to their universal significance. Our analysis builds on English language sources where presentations and discussions of VINITI at international conferences and journals have been the primary material. Russian language sources have supplemented these descriptions – for example, the biography of Nesmeyanov and descriptions of VINITI aimed at a Russian readership – and secondary literature on the organisation of Soviet science. The latter sources are foremost used in the first part of the paper in which the inception of VINITI, and the important role of the Soviet Academy of Sciences are outlined. Then follows the three thematical sections – concentration, abstracting, and pre-printing – each focusing on noteworthy ideas which structured how VINITI was organised and presented. In a concluding section, we revisit the main questions asked above by discussing the wider impact and legacy of VINITI. However, before approaching these issues in detail we briefly describe the context in which VINITI emerged.

## Soviet Science and the Problem of Foreign Literature

Before the establishment of VINITI, the main source for getting access to foreign literature in the Soviet Union was the Moscow Library of Foreign Literature, which was founded in 1921. The library was headed by Margarita Rudomino who managed to keep it open despite the strong anti-foreign campaign starting in August 1946. Her strategy was to emphasise how the library worked against “the penetration of capitalist ideology”, and ultimately a decree from Stalin himself secured the continuation of its operations. (Richards 1998: 202). Still, the library had minimal funds, if any for purchasing foreign literature, and relied on donations from sympathetic foreign socialists and later seizures from German libraries during WW2. After the war the library’s focus on scientific documentation was accentuated and systematic indexes for a range of subjects were produced. These publications were the prototype for the “abstract journals” that became the main activities pursued by VINTI (Richards 1996: 243).

VINITI itself was initiated during what Pollock (2006) describes as the twilight years of Stalin’s rule, and although a less rigid policy on science might have been hinted at, it was still impossible to achieve anything without the support of Stalin. These were years characterised by “utmost secrecy, Cold War paranoia, and spy mania” (Kojevnikov & da Silva Neto 2019: 386). The late forties and early fifties were marked by deteriorating relations with the former Western allies and increasing Russian nationalist sentiments. In 1947, the anti-cosmopolitan campaign was launched, targeting “rootless cosmopolitanism”, foreign influences and servility to the West. Sometimes described as a “war on cosmopolitanism”, the campaign tied in with the emerging Cold War and served to create images of an enemy, although a relatively diffuse one compared to the recent World War II. The anti-cosmopolitan campaign targeted both global imperialism, the “West”, as well as specific groups within the Soviet Union, and especially from 1949 onwards the campaign gained a distinctive antisemitic character (Azadovskii & Egorov 2002; Pollock 2006). Thereafter, the achievements of Russian and Soviet scholars were to be accentuated while the role of Western science was downplayed, and scientists were encouraged to contest or criticise breakthroughs and theories originating from Western countries (Vucinich 1984: 227–237). As Chernyi notes in his history of VINITI, the establishment of the institute at this point in history might seem paradoxical, but he also suggests that the rivalry with the West highlighted the need to be up to date with scientific developments (Chernyi 2005: 23). Aronova (2021: 152) also describes VINITI as a “safe haven” for Jewish scientists that no longer were allowed to continue their careers in universities or research centres. Many of them had good knowledge of foreign languages and came to work at the institute as translators and abstractors.

At the same time, the first decades of Soviet rule had seen a remarkable expansion of science in the Soviet Union. “The characteristically Soviet model of science became established in the mid-1930ies. Sergei Vavilov, the President of the USSR Academy of Science from 1945 to 1951, described its key features, including generous government funding, emphasis on practically useful research, and a structural organisation in which privileged research institutions with large, multidisciplinary teams of scientists, engineers and technicians worked together on the pursuit of goal-oriented research, combining basic science with technological inventions” (Kojevnikov & da Silva Neto 2019: 377). The expansion of science came with increased prestige and science had a privileged status in the post-war Soviet Union. This was reflected not only in salaries but in other perhaps more important ways (as there were many things money could not buy in the Soviet Union) such as access to special shops with superior products, land for dachas (summerhouses/allotment gardens) and bigger and better housing. However, these privileges “came with strings attached” (Kojevnikov & da Silva Neto 2019: 387). The expectations on scientists were high, they were to “catch up and surpass”, as the slogan said, science in the West. Military considerations were something for scientists to take into account, as their research was increasingly expected to have practical applicability.^1^ To be competitive, however, scientists needed fast and efficient access to scientific information, and especially foreign literature.

Hence, VINITI was created at a time of transformation and rapid development of Soviet science. The expectations on future achievements were high, and the grand ambitions of research were evident in the envisioned organisation for handling scientific information on an unprecedented scale.

## Nesmeyanov’s Dream: A “Colossal Enrichment Plant” for Information

The chemist Aleksandr Nesmeyanov replaced the physicist Sergei Vavilov (1891–1951) as president of the USSR Academy of Sciences in 1951. Notably, both Nesmeyanov, and his predecessor Vavilov shared an interest in matters concerning information management, and the problem of scientific information had been on the agenda in Soviet science since the 1930s (Gilyarevskii & Chernyi 2009; Aronova 2021: 137). In his autobiography *Na kachelyakh XX veka* (On the swings of the 20th century) Nesmeyanov describes the creation of VINITI as one of his main achievements, second only to the creation of the new campus of Moscow State University. The purpose of VINITI was to “provide scientists and technicians with exhaustive information on all the achievements in science and technology throughout the world.” (Mikhailov 1959: 513). One additional benefit was that the project would give access to international scientific literature, of which there was a serious deficit, even in Moscow – not to mention in more provincial locations (Chernyi 2005: 22). Nesmeyanov talks about the necessity to make copies of the most important international journals. Such a gigantic scheme would require thousands of qualified workers, and large enough buildings to house the institute. It also required significant allocations of *valiuta* – foreign currency, primarily to take up subscriptions on all these journals. Hard currency was always in short supply in the Soviet Union, so this was a significant issue.

In outlining the background for Nesmeyanov’s own interest in scientific information, he says that it is impossible for a chemist not to be interested in the organisation of scientific literature. From their very early student days, Nesmeyanov and other chemistry students kept records of the literature, and cards with bibliographic information kept heaping up in their hundreds if not thousands. So, the question then arose: how to organise this information so that the right publication could be found when needed? He then describes his own attempt to deal with this problem by organising a searchable system using punch cards (some 20–30 years before these came into standard use in the organisation of scientific information). Later, in a first more organised attempt, an index called “Khimia [Chemica]”, was produced by Nesmeyanov in collaboration with the Academy of Sciences. This became the prototype for the review journals, *referativnyi zhurnaly*, published by VINITI (Richards 1996: 243*)*. In fact, when detailing his early interest in scientific information, Nesmeyanov mentions an attempt at creating an abstract journal for chemistry already in the 1930s, before succeeding in the 1950s when he as president of the Academy of Sciences had the opportunity to return to these questions on a much grander scale, to solve them not only for chemistry but for all (natural) science and associated technical branches.

Nesmeyanov’s background as a chemist is important for understanding his interest in scientific information. It is unmistakeably so that many of the visionaries in terms of organising scientific knowledge came from the field of chemistry. An early example is the famous chemist Wilhelm Ostwald whose work in *Die Brücke* aimed to create “…a central station, where any question which may be raised with respect to any field of intellectual work whatever finds either direct answer or else indirect, in the sense that the inquirer is advised as to the place where he can obtain sufficient information” (Ostwald 1913, cited by Hapke 1999: 142). Similarly, later information entrepreneurs, such as Monty Hyams with his efforts to organise the patent literature, as well as Eugene Garfield, the creator of the Science Citation Index, all had a background in chemistry. Apparently, something made chemists prone to taking an interest in the organisation of scientific information. Without being able to account for the motivations of each of the individuals mentioned above, it is possible to find some more general explanations. The work on the periodic table during the second half of the 19^th^ and the early 20^th^ century, with Russian scientist Mendeleev as a leading figure, had shown how an effective and universal organisation of knowledge could be achieved. At the same time, chemistry was, in the mid-20^th^ century, one of the fastest-growing fields of science, and researchers were looking for ways to navigate the increasing mass of publications. Hence, it was not a surprise that chemists took the lead in the reorganising of scientific communication.

Moreover, Nesmeyanov’s background was reflected in how he described the role of the planned institute. As already quoted, he writes in his memoir how he envisioned the future institute as a “colossal enrichment plant (concentrator)” whose raw material was “all the scientific periodicals of the world” and the process of concentration would result in abstract journals providing scientists with easy access to the latest advances in their fields globally (Nesmeyanov 1999: 54). Given the focus on nuclear technologies at the time when VINITI was founded, the comparison with an “enrichment plant” is perhaps not far-fetched. The idea, and consequent operations of VINITI, were based on the notion of ‘concentration’, and the abstract is undoubtedly a central technique for distilling key insights from a larger mass of scientific text. Moreover, the use of the term “concentrator” is interesting from a communication systems perspective as it refers to “a device in a computer network that collects data from separate low-volume transmission channels and retransmits it over a single high-volume channel” (Merriam-Webster Dictionary). In relation to this definition, ‘concentrating’ relates to ideas of unification, to gather information through one channel, or in this case one centre.

Remembering the creation of VINITI, Nesmeyanov recalls the ideas behind the whole project, and from the beginning the centre was envisioned as something much more than just an abstracting and translation service: “I dreamt that the information that resulted from the abstracting process could be added to the memory of a digital accounting machine [the first models had already appeared] and that it could give out this information structured according to different queries”. According to Nesmeyanov, it was this vision that attracted Georgy Malenkov (1901–1988), who at the time was secretary of the Central Committee and would briefly succeed Stalin as the Soviet Union’s leader in 1953. The aspiration of creating a kind of unified memory, through computers, is recurring in the history of VINITI. Much later, when writing on the topic of the future of scientific information, Mikhailov and his colleagues at VINITI refer to H.G. Wells and his visions of a World encyclopaedia, sometimes likened to a World Brain. The idea of a World Brain was formulated by Wells in a book of the same name in 1938, and it was also presented at a conference on the development of science in 1941. This grand scheme for transforming not only science but society at large was built on two key principles: centralisation and concentration (De Wilde & Somsen 2016). The world encyclopaedia was not envisioned as an assortment of miscellaneous facts, but as “a concentration, a clarification and a synthesis” (Wells 1936: 14). This grand synthesis of knowledge was the main goal of VINITI, and a central method for achieving it was through an extensive abstracting service which would serve both scientists and society.

Lastly, the thoughts of a central, and socialist, planning system for science as laid forward by the British philosopher J.D. Bernal became influential for the visions guiding the building of VINITI. Bernal had, partly based on a proposal by the director of US Science Service Watson Davies, launched the idea of an “Institute of scientific information” (Muddiman 2003). Testimony to Bernal’s influence is his repeated meetings with both Vavilov and Nesmeyanov during his many travels to the Soviet Union, and his paper urging for a centralised information system was commonly referred to in the USSR (Aronova 2021: 137). In short, Bernal’s ideas concerning centralisation and planning resonated very well with the visions of VINITI, and a key concern in his writings was how to make science more efficient. One important approach for achieving this goal – as argued both in the writings of Bernal and in the plans of VINITI – was the production, and translation, of abstracts.

## Centralised Abstraction and the Progress of “World Culture”

With a powerful backer in the Soviet Academy of Sciences, the Institute for Scientific Information did not need to start small.^2^ While the Cold War shaped relations with the West, scientists enjoyed renewed possibilities to be part of the international scientific community. This was an opportunity VINITI took. In the late 1950s, there was also considerable interest in Soviet science, as the launch of Sputnik in 1957 was widely seen as proof of the USSR’s scientific capabilities. In connection, the Soviet Union was perceived as making great strides in managing information (Trembach 2019), and such advances were presented to the world in papers and at international conferences.

For example, in November 1958, information specialists, scientists and librarians gathered in Washington for the *International Conference on Scientific Information.* This conference was part of a series of post-war meetings in which the question of how to handle the increasing amount of scientific information produced throughout the world was discussed. Delegations from numerous countries took part in the conference and among the delegates we find Alexander Mikhailov, since 1955 the director of VINITI. When the 1958 meeting took place, the All-Union Institute of Science and Technical Information was already a huge operation, and its central task, to which most of its efforts were directed, was the translation and abstracting of scientific articles^3^. In placing the scientific abstracts at the heart of a well-functioning system for knowledge dissemination, it relied on a tradition which was established early, both in Europe and pre-Soviet Russia.

The practice of abstracting has a long history as a central feature in the organisation of knowledge and science. Using a broad definition of abstracting which incorporates notes, marginalia, extracts, and summaries Skolnik (1979) traces the method of abstracting as far back as the beginning of writing. Using a more restricted definition we could place the birth of the scientific abstract to the early scientific journals such as *Le Journal des sçavans* and *the Philosophical Transactions of the Royal Society of London*, both established in 1665. These, and many of the similar journals that were launched in the late 17th and early 18th century contained an abstract section where articles from foreign journals were summarised (Skolnik 1979; see also Csiszar 2018). The early 18th century was also the time when the first “abstract journals”, which covered a range of subject areas, including literary works, were launched. Abstract journals became popular, and one of the few studies of the genre found no less than 310 abstract journals being published during the period 1790-1920 (Menzer 1977).

Russia was no exception. Russian journals published abstracts of foreign and domestic scientific papers already in the late 18th century, and periodicals specialised in scientific information appeared in the beginning of the 19th century. Mikhailov et al. (1984: 512) indicates that approximately 50 Russian journals devoted to scientific information were in use from 1800-1917. The effort to disseminate scientific and technological information continued after the revolution, and the first abstract journal, *Reports on Scientific and Technical Works in the Republic*, started in 1920. Hence, a tradition of publishing abstract journals was established in Russia, and later the Soviet Union, when work on abstracting was initiated by Nesmeyanov through VINITI. The major difference was the large scale of this endeavour which aimed for universal coverage not only of scientific literature but also of technological information like patents and technical specifications.

In 1953, a journal of abstracts – *referativnyi zhurnal* – was launched by VINITI with the purpose of covering all sciences in one periodical. A few years later, in 1957, the abstracting service covered 455 000 entries, over 13 different subject areas (Mikahilov 1958: 513). In his 1958 presentation of VINITI, Mikhailov et al. (1984: 511) describes the abstract journal as a “vital tool in research, created by the progress of world culture”. Accordingly, abstracting is viewed as an art in itself and its professionalisation and centralisation are emphasised by Mikhailov (1959: 521) suggesting that:

A task which may be insurmountable for an individual specialist can be coped with successfully by a highly-skilled multifarious scientific body. As we see it, our object is to make the Abstracts Journal into a sort of a scientific forum for intellectual exchange between the scientists of all countries.

The abolishment of divisions, between countries and fields, was part of this grand vision of a socialist internationalism of science. However, the challenges of abstracting were in the late 1950s a topical issue among information scientists and scholarly communication specialists around the world. Indeed, the main products of commercial information services at this time, such as Garfield’s “Documation” (Later ISI) and Monty Hyams’ *Derwent* were abstracting services which reported on the new papers and patents. At the Washington conference in 1958, a whole session focused on the matter. Considerable attention was directed to the Soviet solution to the problem of scientific information in which abstracting was a key method. For such an abstracting system to work efficiently, centralisation was a prerequisite according to Mikhailov. In the discussion at the 1958 conference, he argued that the most important reason for VINITI’s success was the “…concentration within a centralized institute of the specialized personnel and the possibility of using all the data and all the advances of machine engineering available, which are only possible in a centralized service” (National Research Council 1959: 657). Such a system, he argued, made it possible to reach researchers in adjacent fields as discoveries in fields such as biology, metallurgy or chemistry may be relevant for a physicist or vice versa. Centralisation was thus the solution to the problem of specialisation which resulted in a lack of communication between disciplinary fields. Indeed, the separation of related research areas in the literature was seen as a major problem – alongside duplication – which centralisation was supposed to solve. These ideas on the integration of disciplines reflected an increasing focus on the interconnectedness of science in the research policy of the post-war Soviet Union (Josephson 1990: 170).

The British philosopher of science J.D. Bernal concurred with Mikhailov’s view when suggesting “…that we ought to consider that we are building a service system, actually, for world science, and we ought to lay down the foundations for it at this meeting, using as nuclei the ganglias that we have already heard from the Soviet Union, from the United States and other places” (National Research Council 1959: 658). Impressed as early as in the 1930s by the Soviet organisation of science, Bernal was convinced that “small-scale science had already passed and that research itself had entered an industrialised, massive stage” (Kojevnikov 2008: 123). Bernal criticised the current, scattered system for disseminating scientific information which he described as being “an enormous and chaotic structure” (Bernal 1939: 117). Bernal (1939: 119) even went so far as to suggest that the current publication system “…wastes both time and money and is a constant source of irritation among scientists”.^4^ Although abstracting was a key practice for researchers to keep up with the literature, this technique still, according to Bernal “… resulted in an enormous amount of overlapping and gaps in abstracting work, and abstracts themselves have reached an unwieldy size” (1939: 17*).* His solution to duplicates and omissions was a system of several connected centres which together offered an all-encompassing service for scientific information on a global scale.^5^ That the notion of a centralised institute covering all scientific and technological information appealed to Bernal may not come as a surprise, but also other attendees at the 1958 conference, like the U.S. professor of government Waldo Chamberlin, used VINITI as a model for an “International Centre of Scientific Information” (Chamberlin 1959).^6^ Precisely, such a global system is envisioned by Mikhailov in one of the transcribed discussions from the Washington meeting. Mikhailov (1959: 657) describes the scientist seeking information as “[…] a gold prospector faced with the Himalayan jungle and the rock formations of the modern libraries” and emphasises at the same time how today’s science is “organically interconnected”, which makes the organisation of information difficult. Against the background of these challenges, he finds that “…the most effective way of assisting the scientists was in the creation of a large central institute which in its activities would embrace the entire output of national as well as international scientific experience accumulated so far”. He then continued by stating that VINITI had, within five years of its existence, solved the very problem that so many of his Western colleagues were struggling with. In his view, the problem facing science was fragmentation, both in terms of languages and nations and in terms of disciplines and fields. The solution, according to the likes of Mikhailov and Bernal, was international (and socialist) universalism realised through the central handling of scientific information on a global scale.

## The Unpublished Publication: Realising “Bernal’s Plan”

In an article commemorating VINITI’s 25th anniversary in 1977, Arkadyi Chernyi outlines the achievements and activities of the institute by stressing the role of centralisation: “Centralisation is the chief organisational and technological principle in the production of information publications by VINITI” (Chernyi 1977: 4). Furthermore, Chernyi describes a service the institute had started to provide which in hindsight appears as remarkably modern: the deposition of scientific works. The idea of a service where completed research works could be deposited with the institute and thus published outside the traditional academic journals and without peer review, clearly functioned similarly to the pre-print repositories used today. The deposition system was intended to ease the load on scientific journals and to primarily comprise works with a narrow and very specialised readership. Initiated in 1961, the number of manuscripts deposited with VINITI was initially quite low; up until 1967, it totalled only 552 works (Basova & Kuznetsova 1975: 7). However, the system from 1967 onwards reportedly fast gained popularity among Russian scientists, and as of 1977 it contained 26,500 so-called ‘typescripts’ across a network of 49 depository centres (Chernyi 1977: 6). The reason for the increase in popularity was various measures taken in the late 1960s and early 1970s for deposited papers to count as publications, which they initially did not, and from 1971 onwards it came to be seen as quite a widespread new method of publishing narrowly specialised scientific works. Works to be deposited included not only scientific articles and shorter pieces but also monographs and proceedings from conferences (up to approx. 200 typewritten pages) (Basova & Kuznetsova 1975: 7).

As deposition became established as a publication method, the procedure became quite elaborate. Papers deposited with VINITI and four other institutes (for the remaining 44 institutes where papers could be deposited conditions were different) would from 1971 on be counted as publications when an abstract of the paper was printed in the *Referativnyi zhurnal* relevant to the paper’s subject area (Basova & Kuznetsova 1975: 8). Following publication of the abstract, the institute would issue a certificate with the author’s name, the manuscript’s title, and the name and issue of the *Referativnyi zhurnal* where the abstract was published. Authors retained the copyright of the manuscript but were not entitled to any honorarium (ibid.). To handle the manuscript deposited with VINITI, a new department of the institute was created: *Sektor teorii i metodiki deponirovaniya nauchnykh rabot*. Described as “a quite complicated system” (depicted schematically on page 9, Basova & Kusnetsova 1975), the *Sektor* would go through all submitted manuscripts, and then evaluate their scientific quality in dialogue with the corresponding scientific branch of the institute where science editors matched the manuscripts with the relevant abstract journal. The manuscripts that the *Sektor* accepted to be deposited were assigned a number and then prepared for deposition in a way that resembled how manuscripts were prepared for publication. From 1973, the deposited manuscripts were microfilmed (*diamikrokarty*).^7^ According to Basova and Kuznetsova, the deposition of papers should not be seen as a negative evaluation of manuscripts not good enough for publication, but as a method to speed up information and a more rational transmission of information through relevant channels (Basova & Kuznetsova 1975: 12). The average publication time in the form of deposition was in 1975 four months (Basova & Kuznetsova 1975: 9). Basova and Kuznetsova also stress the role of libraries in being able to provide copies of deposited manuscripts in the same way as they provide journals (1975: 12).

The idea of a fast-publishing system which did not rely on journals was not new, but the Soviet system of deposited scientific works was one of the first attempts at implementing such plans on a grander scale. Indeed, this system has, according to Chernyi (1977: 6) implemented “… in practice and on nationwide scale the idea which has become known to foreign scientists and experts as ‘Bernal’s plan’…”. This refers to Bernal’s suggestion of abandoning journals as the primary unit of scholarly communication and rather focusing on the individual paper.^8^ Bernal imagined an international network of “clearing houses” which would handle, index, translate, and distribute individual papers. The building of such a system was initiated already in June 1945. The centralised system, called “the British Publishing Authority” would administer the whole process of publishing and disseminating scientific knowledge. Subsequently, this national system would be integrated into an “International Central Science Library” which was envisioned as a global indexing and abstracting service (Muddiman 2003). The grand vision of such a library was never realised in practice, instead, the idea of a clearing house for scientific papers reappeared under the auspices of VINITI.

While Bernal and his ideas on how to organise science are frequently referred to by representatives of VINITI, it also becomes evident that the independence – and progressiveness – of the Soviet solution were emphasised. Hence, when describing the deposition system, Chernyi notes with pride that this idea “was first advanced by Soviet authors back in 1933” (1977: 6). What he refers to is a discussion piece in the journal *Science* (Goldman 1934) in which a system of “separates” is proposed by Fedorovsky, the president of the Association of Scientific Institutions of the Mining Industry in the USSR, and its secretary Salkind. These “separates” should be distributed by central bibliographical institutes, and this proposal was cited by Bernal himself in his discussion of “clearing houses” (Bernal 1939). Still, Fedorovsky’s and Salkind’s idea of “separates” appears not to suggest the abolishment of journals, but rather that individual articles should be distributed separately if demanded, which is different from Bernal’s vision of abolishing journals altogether.^9^ Regardless of the exact lineage of the idea, the deposition of papers was a radical invention in science communication, an attempt at creating a central “clearing house” for science which operated on a grand scale without any independent publishers or journals.

The deposition of scientific works is perhaps the most striking example of how the socialist solution of a universal and centralised system offered possibilities, which were, at least from a Soviet perspective “impractical for capitalist America” (Chernyi 1977: 6). Indeed, the ideas of copyright and intellectual property were alien to many Soviet scientists, and in a *festschrift* dedicated to Mikhailov it is described how he struggled with the concept: “Professor Mikhailov was initially unfamiliar with some characteristics of Western, and especially US, practices such as copyright. He felt that publication of information within a nation constituted unrestricted right for that nation’s government to freely distribute the information worldwide” (Caponio et al. 1990: 4). Yet, in the 1960s and 1970s the Soviet Union joined international intellectual property instruments, acceding to the Paris Convention in 1967, becoming a member of WIPO in 1967 and joining the World Copyright Convention in May 1973. The recognition of copyright had consequences for VINITI, which had to discontinue the so-called Express information service. These were bulletins consisting of extensive abstracts, or rather abridged translations of important foreign publications, which made reading the original publication unnecessary. Yet, this practice which according to Chernyi (1977: 4) “…made a great contribution to surmounting language barriers”, was not acceptable practice according to international copyright agreements and had to be abandoned.

## Discussion: The Influence and Legacy of VINITI

In brief, VINITI was created to solve the concrete and crucial problem of assessing up-to-date scientific information in Soviet society. At the same time, the visions of VINITI echo of a European tradition of grand projects to unify knowledge which can be likened to similar “World projects” such as Ostwald’s “die Brucke”, Otlet’s “Mundaneum” and Wells’ “World Encyclopedia” (Krajewski 2015). In contrast to these projects which were all based on the grand visions of its founders, VINITI was initiated by a totalitarian state and the handling of information was part of efforts to centrally steers society at large. VINITI had its visionaries – first Nesmeyanov and later Mikhailov – and its creation can partly be viewed in relation to previous efforts to provide scientific literature to Soviet scientists through libraries and information services. Still, the sheer ambition and magnitude of the project reflected that this project was something else. It was designed and presented as the ultimate solution to the problem of scientific information; a problem that scientists and librarians had been grappling with for decades.

The institute, which was intended to serve Soviet needs, had global ambitions, and it aligned well with Somsen’s description of “socialist internationalism” as a specific form of scientific universalism. An ideology in which science (in socialist form) would direct government decisions on all levels while at the same time abolish divisions between countries, and classes (Somsen 2008). A view that both Wells and Bernal adhered to in their writings on how science (and socialism) would transform society. Important for this view on universalism was the idea that science was not the product of a particular cultural context (e.g. Western Europe) but rather “…a universal instrument to solve practical problems” (Somsen 2008: 372). These views resonate with the strong belief in translation – and often machine translation – in the Soviet handling of scientific information. The loss of cultural, and linguistic, meaning would not be a problem if science is viewed as a universal tool, rather than a practice which is constantly formed by its context. This socialist view of universalism is moreover evident in the coverage of scientific literature, where VINITI, contrary to many of its Western competitors, offered a geographically broad coverage of countries and languages. However, as shown above, scientific universalist ideals were always threatened by tendencies towards isolationism and repression, and the needs of scientists were balanced by ideological motives and political campaigns. At the same time, it is obvious that political leaders – even Stalin – recognised the need for an organised and reliable system for providing Soviet scientists with information.

The Soviet solution to the problem of scientific information was ambitious and monumental in terms of the resources and personnel involved.^10^ Through rational centralisation of resources, VINITI would – according to its creators and admirers – achieve an effective and all-encompassing coverage of scientific information serving society at large. By gathering all relevant information and then distilling it into its most potent and pure form, the Soviet solution would – according to its creators – surpass the inefficient and decentralised system used in the West. In fact, VINITI was only one part – albeit central – of a whole system for the exchange and dissemination of information across all parts of the Soviet Union. In our analysis, two key principles emerge as fundamental for its operations and that is the notion of “concentration” and the importance of centralisation. The idea of concentrating knowledge is evoked by Nesmeyanov already at the inception of the institute and many of its key functions – in terms of abstraction, translation, and summarising – involve some form of distillation in which a mass of information is reduced to its most relevant or significant parts. Nesmayanov’s idea of a “colossal enrichment plant” is illustrative of the confidence in an efficient concentration of knowledge, both in the form of one centre that would gather all relevant information, and in the strong focus on the abstract as a method for condensing scientific findings. A fast increase in the amount of scientific and technical information – which accelerated after the Second World War – encouraged scientists and information specialists, like Bernal, to look for, in the words of Nesmenyanov, ways to enrich this vast amount of information into its most valuable bits. Indeed, Bernal’s arguments for “centralisation” – as an alternative to scattered separate initiatives in capitalist countries – was important, and attempts to realise “Bernal’s plan” were made through a system for “pre-publication” of scientific papers.

Approaches for best achieving a distillation of knowledge through various techniques such as annual reviews, indexes, and journals were an item for much discussion throughout VINITIs existence. The abstract journal, with translated abstracts of current literature, did, however, remain the primary service provided by the centre. Abstracting was considered as a fundamental technique for handling information in the early fifties, and commercial services offered a similar solution for handling the increasing amounts of scientific information. Yet, while abstracting soon was replaced by other approaches in the West – for example, citation indexing and online databases for direct searching – the role of the abstract journal remained central for the operations of VINITI. The reliance on this time-consuming technique thus soon made the Soviet system slow, and ineffective, in comparison to Western alternatives.

Hence, in hindsight, VINITI’s emphasis on an all-encompassing, highly bureaucratic, centralised system seemed, like the Soviet Union itself, doomed to fail. Centralisation and bureaucratisation became major obstacles to a competitive information service. American information specialists invited to visit VINITI reacted to the sheer number of people involved, the bureaucratic structure, and the inferior technological equipment used. Eugene Garfield said, in a speech at VINITI’s 50^th^ anniversary in 2002, that the naming of his institute the *Institute of Scientific Information* (ISI) was “a blatant youthful challenge” to VINITI (Garfield 2002). Eventually, however, the Soviet alternative was not competitive with Western solutions, and the sociologist of science Derek de Solla Price commented in 1975 that the Soviet system had become “an unwieldy, disorganised system” and he noted that Soviet scientists instead used American services, such as Chemical Abstracts (Price 1975:182-183). Tellingly, in the 1970s and 1980s, VINITI and the Soviet Union became one of the most important foreign subscribers for the Science Citation Index produced by Garfield and his institute (Aronova 2021: 154).

Still, we argue that the history of VINITI is important if we want to understand the development of scientific information systems after World War II. At the time, it offered a vision – and a practical example – of how a truly centralised system could be envisioned. Hence, by suggesting an alternative and de-universalised (Aronova 2021) history, it may provoke reflections regarding the historical, as well as contemporary, attempts to manage scientific information. What becomes obvious is that the Soviet solution was not an isolated experiment, but an integrated effort which was perused in dialog with Western initiatives. During the 1950s, news regarding “the World largest scientific library” attracted interest from information specialists who grappled with the “information crisis” in their respective home countries. Clearly an interest in the operations of VINITI from Western specialists, and an exchange of views – for example, on the best use of abstracts – can be found in the literature, and several visits by information experts to the Soviet Union are documented (Trembach 2016). For example, early success of ISI in publishing abstracts through the publication of *Current contents* (1955) much resembled the operations of VINITI and its major journal *referativnyi zhurnal* (1952). However, we should not overemphasise the influence of VINITI on Western initiatives in scientific information. Rather, the influence of VINITI may be found in its radical alternative to an increasing commercialisation of scientific information, in which bibliographical information on, and access to, scholarly publications and patents became commodities in a new industry focused on scientific information. In this context, VINITI offered an alternative route in which copyright was deemed as less important than universal access to information. At the same time, the Soviet solution to the scientific information problem had unique features which could not easily be adopted in other contexts, such as the exclusion of private commercial interests and the disregard of copyright. The perhaps most striking invention, to a contemporary reader, is the development of centralised databases that indexed so-called “typescripts”. If not the first, this is undoubtedly one of the earliest attempts of creating a non-journal-based system for the dissemination of scientific papers that much resemble pre-print repositories of today.

The legacy of VINITI can also be described in terms of its training of thousands of science librarians. The institute was for many years a major contributor to library science in which advanced research on the organisation and structure of scientific knowledge was produced. Most notable is perhaps the field of “naukometri” which was later translated into “Scientometrics”, a field to which Soviet researchers made considerable contributions (Cherny and Gilyarevsky 2001). Hence VINITI’s leading figures were active and visible within the larger community of experts on scientific communication. Information scientists from VINITI took a prominent role in establishing scientific information as a research field, and the term “informatics” for describing this area of inquiry was coined by Mikhailov in 1966 (Foskett 1970). A further illustration of the influence of VINITI is its prominent role in the *Fédération internationale de documentation*, later the *International Federation for Information and Documentation*. Indeed, Paul Otlet and his legacy played an important role in the Soviet Union. The classification scheme developed by the International Institute of Bibliography in Brussels, the Universal Decimal Classification System (UDC), was adopted by the Communist rulers already in 1920, and Otlet was held in high regard in Soviet information science (Delougaz 1947).

Today, the main office of VINITI is located in Ulitsa Usiyevicha, 20, in the north-western part of Moscow. It is still in operation, although in a more modest way compared to the twenty thousand people that it employed in the 1960s and 1970s. Reflecting on VINITIs role in the 21^st^ century, a former employee suggested that the centre now is a “surrogate of itself” which serves an increasingly small number of subscribers (Shamaev 2011). In a rejoinder to Shamaevs article, Vladimir Borschev (2011), describes how VINITI was a “truly useful institution” until the 1980s but the arrival of the Internet made its operations outdated. Overall, attempts to re-direct the purpose of VINITI in the online post-Soviet era has so far been largely unsuccessful, although an observer like Borschev (2011) imagines that it might have a potential to serve “practising scientist” in Russia.

In 2022, VINITI celebrated its 70^th^ anniversary in a political atmosphere that in some ways resembles the one in which it was founded, where Russian science is again isolated, international contact and cooperation is hard to maintain and the authorities are very cautious of all things foreign. The information environment, on the other hand, is of course radically different from 1952. The website of the centre, now named the All-Russian Institute of Scientific and Technical Information of the Russian Academy of Sciences (VINITI RAS), describes that its purposes are to conduct research in information science, and to produce “a unified abstract journal”.^11^ Little information about its grander history can be found on the website, and it is largely forgotten in the European history of science and scholarly communication. Yet, during the 1950s and 1960s it offered a radical alternative, which attracted considerable attention from foreign colleagues, for how to organise an effective system for handling the problem of “the information flood” or “information explosion”. In conclusion then, the ambitions and operations of VINITI may today appear as both old fashioned (in focusing on abstracts), bordering on the utopian (in the idea of total coverage), and as visionary and modern in its abandonment of journals and traditional forms of peer review.

## Footnotes

1 This placed demands of secrecy on researchers and limited what they could publish where. As Kojevnikov (2004) points out, the increase in both prestige and importance that science and scientists saw at this time drew them closer to the party apparatus and its ideological demands. Scientific disputes took on an ideological (and bureaucratic) dimension, and both small and large events were staged to critically discuss selected works in which the failing authors were forced to repent and atone for mistakes. The dispute was settled by voting, and once settled was not to be reopened. The sessions were “closed with a single definitive resolution, even though at the initial stages pluralism and freedom had been encouraged” (Kojevnikov 2004: 215). Hence, in the years preceding the establishment of VINITI, Soviet science was characterised by ideological debates and campaigns. The best known of these is the Lysenko affair which, after a session in 1948, effectively banned all neo-Darwinist (genetic) research in the Soviet Union for 16 years. However, this was not the sole debate, as ideological discussions were characteristic of this period. In these debates, the Academy remained an important institution, but at times, such as in the Lysenko case, was also side-lined.

2 The Academy of Science was a powerful and influential organisation. It was inherited from a pre-revolutionary past: it was in fact founded well before any Russian university. Historically, the Academy had great influence on the development of scientific institutions in Russia, and it became further institutionalised in the Soviet period and had an important role in the organisation of Soviet science (Vucinich 1984; Graham 1993a, 1993b: 18f.).

3 However, not all documents handled by VINITI consist of periodical literature. Another important category for scientific and technical information was patents, and these were handled by a special “Patents group”. In 1964, the *All-Union Collection of Standards and Technical Specifications* (VIFS), the *All-Union Institute of Technical Information Classification and Coding* (VNIIKI) as well as the *Central Institute of Patent Information* (CNIIPI) were created in order to coordinate the distribution of more technological information. Indeed, VINITI’s role in the handling of patents – and certificates – was primarily by providing abstracts (Sviridov 1967).

4 Notably, Bernal also questioned the sheer number of journals and articles: “The very bulk of scientific publications is itself delusive. It is of very unequal value; a large proportion of it, possibly as much as three-quarters, does not deserve to be published at all, and is only published for economic considerations which have nothing to do with the real interests of science” (Bernal 1939: 118).

5 Bernal (1939: 302) further described how such a system would operate: “Each distributing centre in a national area would deal with papers written in the national language. The editorial board would arrange that all papers adjudged of sufficient importance, together with those for which there is special demand abroad, should be translated into one or all of the three scientific languages”.

6 In outlining an “International Institute for Scientific Documentation” Chamberlain specified the annual budget for such an organisation to $283,350,000 (Chamberlin 1959: 1532).

7 Copies of deposited papers could be requested (against a fee) as xerox, photocopies or microfilm. In 1972, 3,857 manuscripts were requested for copy, and in 1973 the number was 7,029 manuscripts. Copies should be received within one month of the request (Basova & Kuznetsova 1975: 9-10).

8 Bernal claimed that the only reason why journals were not already abolished was nostalgia and “purely sentimental reasons”. He then states that “After all, periodicals exists for science and not science for periodicals” (Bernal 1939: 300).

9 The idea of “separates” appears to be a development of an older tradition of circulating so-called “separate copies” or “offprints” (see Spoerhase 2020).

10 An illustration of the ambitions and resources available are the early plans of a private VINITI airplane that would guarantee swift import of foreign periodicals (Chernyi 2005: 24).

11 http://www.viniti.ru/ (accessed 2024-01-08)
